# Hematopoietic Stem/Progenitor Cell Sources to Generate Reticulocytes for *Plasmodium vivax* Culture

**DOI:** 10.1371/journal.pone.0112496

**Published:** 2014-11-13

**Authors:** Florian Noulin, Javed Karim Manesia, Anna Rosanas-Urgell, Annette Erhart, Céline Borlon, Jan Van Den Abbeele, Umberto d'Alessandro, Catherine M. Verfaillie

**Affiliations:** 1 Unit of Malariology, Institute of Tropical Medicine, Antwerp, Belgium; 2 Department of development and regeneration, Stem Cell Institute, Leuven, Belgium; 3 Unit of Veterinary Protozoology, Institute of Tropical Medicine, Antwerp, Belgium; 4 Medical Research Council Unit, Fajara, The Gambia; Instituto de Higiene e Medicina Tropical, Portugal

## Abstract

The predilection of *Plasmodium vivax* (*P. vivax*) for reticulocytes is a major obstacle for its establishment in a long-term culture system, as this requires a continuous supply of large quantities of reticulocytes, representing only 1–2% of circulating red blood cells. We here compared the production of reticulocytes using an established *in vitro* culture system from three different sources of hematopoietic stem/progenitor cells (HSPC), i.e. umbilical cord blood (UCB), bone marrow (BM) and adult peripheral blood (PB). Compared to CD34^+^-enriched populations of PB and BM, CD34^+^-enriched populations of UCB produced the highest amount of reticulocytes that could be invaded by *P. vivax*. In addition, when CD34^+^-enriched cells were first expanded, a further extensive increase in reticulocytes was seen for UCB, to a lesser degree BM but not PB. As invasion by *P. vivax* was significantly better in reticulocytes generated *in vitro*, we also suggest that *P. vivax* may have a preference for invading immature reticulocytes, which should be confirmed in future studies.

## Introduction

*Plasmodium vivax* (*P. vivax*) is the most widespread malaria parasite outside sub-Saharan Africa, and accounts for 80 to 300 million of malaria cases per year [1]. The predilection of *P. vivax* for reticulocytes is a major obstacle for the establishment of a long-term *P. vivax* culture system [2], [3]. As reticulocytes represent only 1–2% of the circulating red blood cells (RBCs) with a half-life of 2 days (including 1 day in the peripheral blood), collecting sufficient reticulocytes to maintain a *P. vivax* culture is a challenge [3].

It has been previously shown that reticulocytes can be obtained by concentrating adult peripheral blood (PB) or umbilical cord blood (UCB) using either a 70% percoll solution [4] or a plasma autologous ultra-centrifugation [5]. One study suggested that *P. vivax* could be maintained in culture for up to 85 days with reticulocytes concentrated from umbilical blood cord (UCB) [6]; however, parasites did not develop beyond one schizogony cycle and parasite densities were very low [7]. In addition, it is possible to culture hematopoietic stem/progenitor cells (HSPC)/CD34^+^ cells to induce erythroid differentiation and consequently produce reticulocytes *in vitro* [8]. Reticulocytes generated from CD34^+^ cells from both bone marrow (BM) and peripheral blood mononuclear cells (PBMC) have been previously used for culturing *P. vivax*; but the authors did not provide data regarding the efficiency of invasion and the development of the parasites *in vitro* [9].

In this report, we compared different sources of hematopoietic stem/progenitor cells (HPSC), namely UCB, BM and peripheral blood, for their capacity to produce reticulocytes that allow invasion by *P. vivax*.

## Materials and Methods

### HSPCs expansion and reticulocytes differentiation

#### CD34^+^ cell isolation

The differentiation of HSPC into reticulocytes was done according to a previously described protocol [10]. UCB was obtained from the Belgian Cord Blood Bank at the Gasthuisberg Hospital Leuven, BM samples were obtained from volunteer donors; and human peripheral blood from the Antwerp Red Cross. Mononuclear cells from peripheral blood (PBMC), UCB and BM were isolated on a Ficoll gradient (GE Healthcare), by 30 minutes centrifugation at 400 g. The mononuclear cells were collected and washed twice with PBS. CD34^+^ enriched HSPCs were isolated using Magnetic Assorting Cell Sorting (MACS, Biotenyl Biotech). HSPC cell purity after MACS selection was assessed by FACS analysis, using CD34 and CD45 antibodies (eBioscience).

### Reticulocytes differentiation

CD34^+^-enriched cells were dispensed in a 6-well plate with IMDM medium (Gibco) supplemented with L-glutamine (4 M, Sigma), penicillin-streptomycin (1%, Invitrogen), folic acid (10 µg/mL, Sigma), inositol (40 µg/mL, Sigma), transferrin (120 µg/mL, Sigma), monothioglycerol (1.6 10^−4^ M, Sigma), insulin (10 mg/mL, Sigma) and 10% human plasma. During the first 8 days, the medium was supplemented with the following factors: hydrocortisone (HDS, 10^−6^ M, Sigma), interleukin-3 (IL-3, 5 ng/mL, R&D system), stem cell factor (SCF, 100 ng/mL, Bioke) and erythropoietin (EPO, 3 IU/mL, R&D system) and placed at 37°C in a 5% CO_2_ incubator. The initial volume of medium was 4 mL and after 4 days, an extra 3 mL was added. After 8 days, the cells were centrifuged for 5 minutes at 300 g, fresh IMDM medium supplemented with EPO (3 IU/mL) was added, and the cells were transferred in a 25 cm^2^ flask. On day 11, the medium was changed and complete medium was added without EPO. Afterwards, medium was refreshed every 3 days and 10% heat inactivated human serum was added to protect the viability of the cells.

For CD34^+^ cell expansion, CD34^+^ enriched cells were dispensed in a 6-well plate with 4 mL Serum-free expansion medium (SFEM, Sigma) with SCF (50 ng/mL), thrombopoietin (TPO, 50 ng/mL, R&D system), FMS-like tyrosine kinase 3 (FLT3, 50 ng/mL, R&D system) and IL-6 (50 ng/mL, R&D system) for 5 days at 37°C, and 5% CO_2_. On day 5, the cells were counted, and transferred into a new 6-well plate to induce the reticulocyte differentiation (using the protocol described above).

### Reticulocyte concentration

Reticulocytes were enriched from UCB or PB by loading on a 70% isotonic percoll cushion which was spun for 15 minutes at 400 g. After two washes with PBS, reticulocytes were counted as described below.

### Reticulocyte count

Cells were spun at 300 g for 5 minutes and re-suspended in 50 µL of PBS; 50 µL of Cresyl blue (Roche) diluted 1∶1000 was added and cells were incubated at room temperature for 30 minutes. After a cytospin centrifugation (3 minutes at 700 rpm), the cells were fixed with methanol, and stained for 10 minutes with Giemsa (Sigma). The slides were then examined by microscopy (immersion objective, 630× magnification), and reticulocytes were counted against a minimum of 500 RBCs and the density per 100 RBCs was computed. A reticulocyte was morphologically defined as an enucleated cell with at least 3 dots of cresyl blue RNA.

### *Plasmodium vivax* invasion assays

Cryopreserved *P. vivax* isolates [11] from infected patients were provided by the Shoklo Malaria Research Unit (SMRU, Mae Sot, Thailand). The samples were thawed with NaCl solutions and cultured for 36 to 40 hours with McCoy's medium (Gibco) supplemented with glucose (2%) and 20% heat inactivated human serum. *P. vivax* mature forms were concentrated on a 45% percoll after a 5 minutes treatment with 0.05% trypsin. After 15 minutes of centrifugation at 1600 g, cells above the 45% percoll were collected and washed twice before checking the quality of the concentration. If more than 90% of the cells contained parasites, they were mixed with our previously differentiated and cryopreserved reticulocytes (chosen to contain the same percentage of reticulocytes for all the conditions tested) in a 96-well plate and the initial parasite density was adjusted on a 1∶6 ratio (final volume 100 µL, hematocrit 2–5%). Cells were checked at 24 hours post-invasion by doing a cytospin slide stained with Giemsa. The parasite densities were computed after examining a minimum of 500 RBCs.

### Data analysis

Data were entered and analyzed with STATA12 (StataCorp, Texas). Reticulocytes were counted after 14 days of differentiation and the mean±SD calculated for each source of HSPC. The Kruskall-Wallis test was used to compare population means. Means and standard deviations were calculated to summarize HSPC expansion rates.

### Ethics statement

*P. vivax* samples collection was approved by the ethics committees of the faculty of tropical medicine, Mahidol University, Bangkok, Thailand (number MUTM-2008-15) and the University of Oxford, Centre for Clinical Vaccinology and Tropical Medicine, United Kingdom (Ethics approval number: OXTREC 027-025). UCB were collected from the cord blood bank at the Gasthuisberg Hospital, Leuven, Belgium (Ethics approval number ML6620). Bone marrow samples were taken from voluntary donors at the Gasthuisberg hospital, Leuven, Belgium (Ethics approval number B322201112107). Adult peripheral blood samples were bought from the Antwerp Red Cross blood bank.

A written inform consent was signed by each donor.

## Results

### Reticulocyte production from BM, PB and UCB CD34^+^-enriched cell populations

Reticulocyte differentiation was successfully induced from magnetically sorted CD34^+^-enriched populations from UCB, PBMC and BM in three independent experiments (n = 3). The enrichment for CD34^+^ cells in the sorted populations was 55% (SD±6) for UCB, 35% (SD±8) for BM and 16% (SD±6), for PBMC (3 independent experiments) as determined by FACS. The peak of enucleation occurred after 14 days of differentiation, regardless of the source. The enucleation of erythroid cells from PBMC (mean = 32, SD±6) was significantly higher (p = 0.002) than that of UCB (18%, SD±1.3 and BM (21%, SD±1.5) (Table 1, 6 independent experiments).

**Table 1:** Hematopoietic stem progenitor cells (HSPC) expansion and reticulocyte differentiation for three different sources HSPCs (6 independent experiments were carried out for each HSPC source).

HSPC sources	Mean proportion (%) of reticulocytes at D14 (± SD) (n = 6)	Cell number mean fold increase (± SD) (n = 3)	
		After 5 days of expansion	After 7 days of differentiation
UCB	18.3±1.3	11.5±2.3	33.5±2.4
BM	20.5±1.5	3.1±0.3	8.6±0.5
PBMC	32±6	1.3±0.2	3.4±0.2

Results show the mean proportions of reticulocytes observed at the peak of enucleation after 14 days of culture, as well as the increase in total cell number after 5 days of expansion and 7 days of differentiation (3 independent experiments). After 5 days of expansion, the number of cells was counted and divided by the initial number of plated MACS/CD34^+^ cells, while after 7 days of HSPC differentiation, the cell count was compared to the number of cells not previously expanded, results are expressed in mean fold increase (± SD).

### Reticulocyte production from *ex vivo* expanded BM, PB and UCB CD34-enriched cell populations

**Figure 1:**
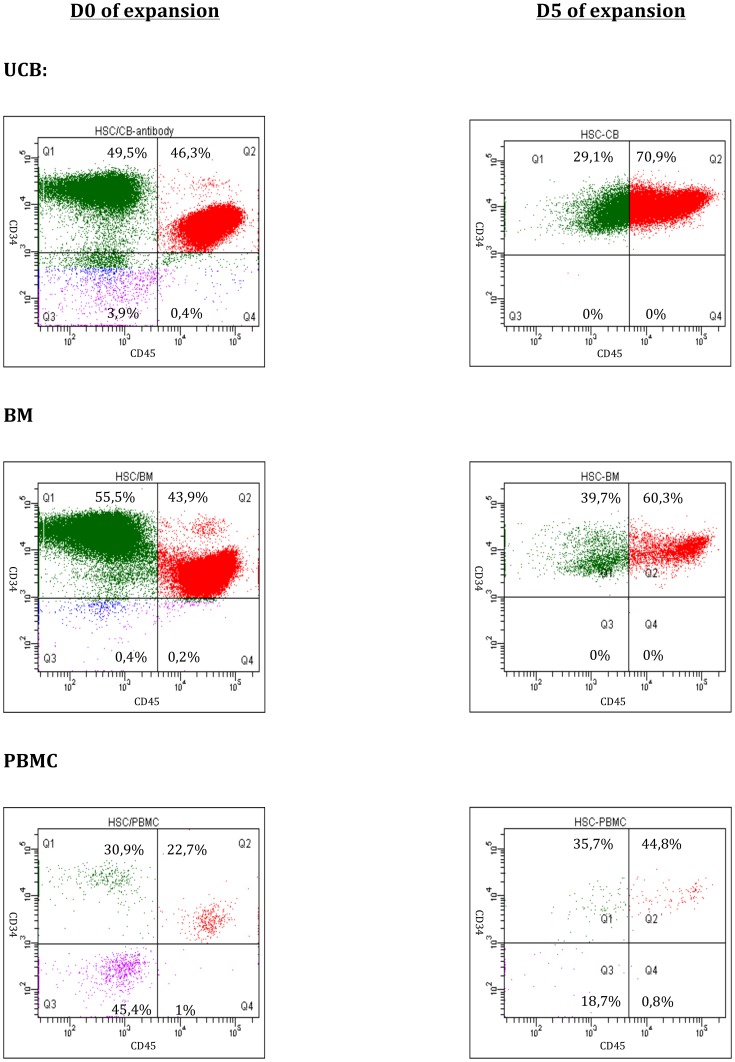
FACS analyses of the CD34^+^/CD45^+^ cells from UCB, PBMNC and BM, after isolation (Day 0) and following 5 days of expansion. The Q2 gate represents the population double positive for CD34 (APC) and CD45 (PE).

We next tested if larger numbers of reticulocytes could be obtained from *ex vivo* expanded CD34^+^-enriched cell populations. After 5 days of expansion in serum-free medium with TPO and SCF, the total cell populations increased >10-fold in cultures initiated with UCB/CD34^+^-enriched cells, 3-fold for BM/CD34^+^-enriched cells while for PBMC no expansion was observed (Table 1, 3 independent experiments). FACS analysis demonstrated an increase in the CD34^+^/CD45^+^ population between Day 0 and Day 5 for all three cell sources: from 55% to 70% (SD±2) for UCB, 35% to 55% (SD±5) for BM, and 16% to 29% (SD±16) for PBMC (n = 3 for CB and BM, n = 2 for PBMC)(Figure 1).

**Figure 2:**
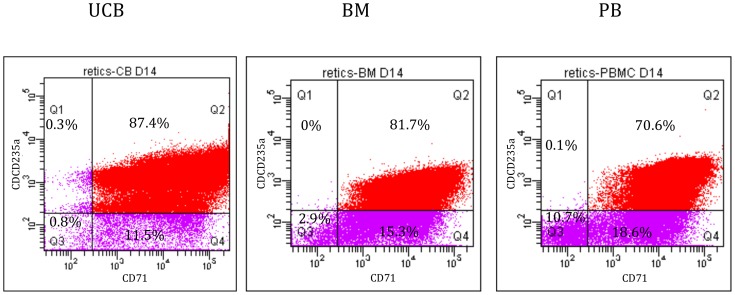
FACS analyses of the CD235a^+^/CD71^+^ cells from UCB, PBMNC and BM, after 5 days of expansion and 14 days of differentiation. The Q2 gate represents the population positive for CD235a (Per-CP-Cy5-5) and CD71 (PE).

Following expansion, a similar number of cells (irrespective of the CD34^+^ content or expansion) were cultured under reticulocyte differentiation conditions. After 7 days of erythroid differentiation, the total number of cells, previously subject to an expansion step, was 3 times higher compared to CD34^+^ cells that were immediately induced to differentiate. After 14 days of differentiation, expanded cells expressed high levels of CD235a and CD71 receptors, regardless of cell source (respectively 87.4% for UCB, 81.7% for BM and 70.6% for PB; Figure 2). Compared to unexpanded cells, the proportion of reticulocytes obtained at day 14 from *in vitro* expanded CD34^+^ cells was 5 to 10-fold higher.

### *P. vivax* invasion

**Figure 3:**
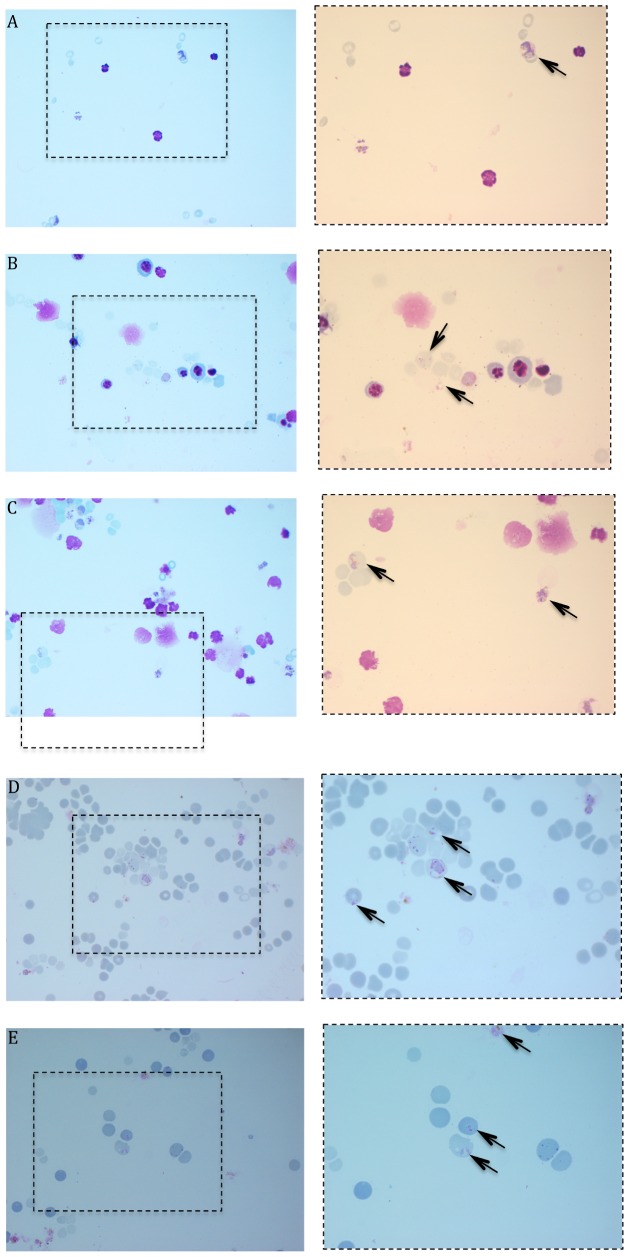
Cytopsin of the *P. vivax* culture 24 h post-invasion for different sources of reticulocytes. A) UCB/HSPC, B) BM/HSPC, C) PB/HSPC, D) reticulocytes-enriched UCB and E) reticulocytes-enriched PB. The left panels represent pictures with a 63× magnification and the corresponding right panels represent a 100× of the left picture square. *P. vivax* infected cells are under arrow.

*P. vivax* parasites invaded reticulocytes derived from CD34^+^ cells or directly obtained from enriched blood (PB or UCB; Figure 3). The mean invasion rates for the different sources of HSPC sources were 3.05%, 3.05% and 3.15% respectively for UCB, BM and PB (n = 4). The means for UCB-concentrated reticulocytes (n = 4) and PB-concentrated reticulocytes (n = 3) were respectively 1.4% and 0.2%.

**Figure 4:**
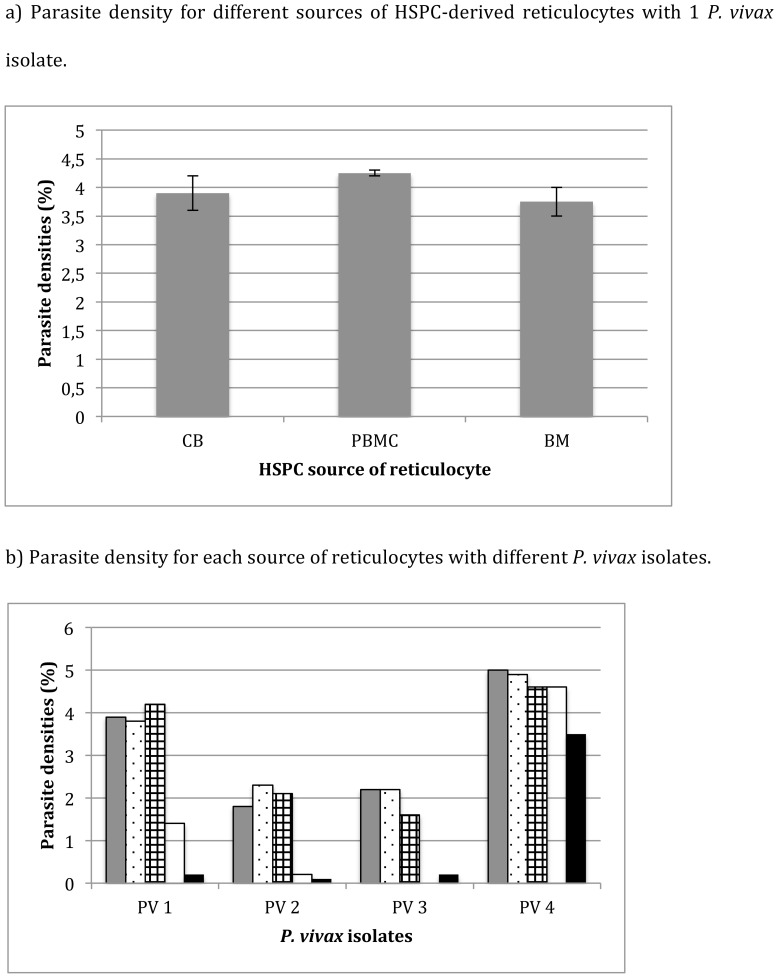
Parasite densities 24 hours post-invasion with *P. vivax*. The parasite density was counted for at least 500 red blood cells, dividing the number of infected enucleated cells by the total number of cells and multiplied by 100 (%). a) Parasite density for different sources of HSPC-derived reticulocytes with 1 *P. vivax* isolate. The mean and SD of 2 different batches of differentiated reticulocytes was calculated for each source of HSPC and tested for invasion with the same *P. vivax* isolate. b) Parasite density for each source of reticulocytes with different *P. vivax* isolates. Parasite densities (%) were counted by dividing the number of *P. vivax* ring-infected cells by the total number of counted RBCs and multiplying the result by 100. Different reticulocyte sources were tested: grey  =  UCB/HSPC-derived reticulocytes; dotted  =  BM/HSPC-derived reticulocytes; squared  =  PBMC/HSPC-derived reticulocytes; white  =  reticulocytes concentrated from UCB, black  =  reticulocytes concentrated from adult peripheral blood. PV1 and PV2 were tested with the same batches of HSPC-derived reticulocytes for the 3 different sources (UCB, BM and PBMC). For PV4, the proportion of reticulocytes was 20% for HSCP-derived reticulocytes and respectively 70% and 60% for reticulocytes concentrated from UCB adult peripheral blood.

When using the same *P. vivax* isolate, the invasion rate between different HSPC sources did not differ for all of the 4 *P. vivax* isolates tested (Figure 4a); however, the invasion rate observed varied for each of the *P. vivax* isolates used. When we compared two different *P. vivax* isolates using the same HSPC-derived reticulocytes, the invasion rate varied significantly by isolate (Figure 4b; PV1 and PV2, p<0.001). After 3 days of culture, only few rings (parasite density <0.05%) could be observed and none survived longer than 72 hours, regardless of the HSPC source. Interestingly, the invasion rate of *P. vivax* in HSPC-derived reticulocytes appeared to be higher when compared with reticulocytes isolated directly from PB. For the same *P. vivax* isolate, the parasite density 24 hours post-invasion in UCB/HSPC-derived reticulocytes was up to 9-fold higher than in UCB-concentrated reticulocytes (1.8% *versus* 0.2%, respectively), and 18-fold higher than adult PB-concentrated reticulocytes (2.1% *versus* 0.1% respectively). Parasite densities were not significantly different between HSPC-derived reticulocytes (5%), UCB-concentrated reticulocytes (4.6% p = 0.056) and PB-concentrated reticulocytes (3.5% p = 0.06) when the reticulocyte percentage was 20% for HSPC-derived reticulocytes and respectively 60% and 70% for reticulocytes concentrated from PB and UCB.

## Discussion

In this study, we compare for the first time different source of HSPC to generate reticulocytes suitable for *P. vivax* studies. We could demonstrate that compared with CD34^+^-enriched populations from PB and BM, CD34^+^-enriched populations from UCB produced the highest number of reticulocytes that can be invaded by *P. vivax*. Second, when CD34^+^-enriched cells were first expanded, a further extensive increase in reticulocytes was generated from UCB, to a lesser degree BM but not PB.

The number of reticulocytes derived from UCB CD34^+^ enriched cell populations could be substantially increased when the CD34^+^ cells were first expanded for 5 days. Noteworthy, in our experiments PB/HSPC cells showed a limited increase of the population after 5 days of expansion compared to UCB/HSPC and BM/HSPC. This is likely due to the low frequency of CD34^+^ cell in non-mobilized PBMC [12] and the more mature fate of HSPC in PB or BM compared to cells from UCB [13].

Reticulocytes derived from magnetically sorted CD34^+^ cells from either PBMC, BM or UCB could be invaded by *P. viva*x with similar efficiencies, while invasion was significantly influenced by the type of *P. vivax* isolate. Despite successful invasion, none of the produced reticulocyte population could support the full development and long-term culture of *P. vivax*. Reticulocytes generated in this study were more permissive for *P. vivax* invasion compared with the study published by Panichakul *et al* [6], who observed only 0.0015% of invasion, mainly due to a very low percentage of reticulocytes (0.5%); or Furuya *et al* [14] who used frozen erythroblast derived from UCB/HSPC (0.8% parasitemia). The 3.5% parasitemia rate observed in our study are in line with the invasion rate observed by Borlon *et al* (2.1%) [11] and Russell *et al* (3.7%) [4], both using reticulocyte-enriched UCB (percentage of reticulocytes greater than 50%).

Our observations might also suggest a preference of *P. vivax* for more immature reticulocytes as we observed a higher invasion rate of reticulocytes derived from any HSPC source compared to those concentrated from either UCB or PB. The higher invasion rate in HSPC derived reticulocytes concentrated from UCB compared to those from PB could be explained by the distribution of their reticulocytes populations. Indeed, Paterakis *et al* [15] classified reticulocytes according to their RNA content by FACS analysis and divided them into 3 categories, i.e. immature reticulocytes (high amount of RNA), median, and old reticulocytes (medium and low amount of RNA, respectively). They found that among the reticulocyte population, UCB contains more immature reticulocytes (13.6%) than adult peripheral blood (1%). Furthermore, HSPC-derived reticulocytes were collected at the peak of enucleation, i.e. when they were at an immature stage of development. This provides additional evidence for preference of *P. vivax* for immature reticulocytes and should be further investigated.

When reticulocyte-enriched blood from UCB and PB was used at 3-fold higher reticulocyte concentrations (70% and 60%, respectively), the invasion rates became similar to that obtained with HSPC-derived reticulocytes (20% for HSPC). This is in line with a recently published report by Martín-Jaular *et al* [16] wherein the authors observed a predominant invasion of CD71^high^-expression cells (CD71 being a marker of reticulocyte maturation, as their expression decrease while the reticulocyte maturate to the RBC stage) by *P. yoelii* (a mouse *Plasmodium* species also invading preferentially reticulocytes).

Our preliminary observations need further in-depth investigation to provide new insights into the invasion mechanisms of *P. vivax*, and more specifically on the critical stage-specific receptors. If confirmed, this hypothesis would justify the use of reticulocytes derived from HSPC instead of reticulocyte-enriched blood as target cells for the establishment of continuous *P. vivax* cultures.

In conclusion, our results demonstrate that it is possible to produce large amounts of immature reticulocytes that can be efficiently invaded by *P. vivax*. The ability to derive reticulocytes from UCB/HSPCs in in larger quantities than from PB or BM/HSPCs without loosing the permissiveness to *P. vivax* make this source of HSPC as more suitable and likely to develop into a standardized and continuous source of reticulocytes for the long-term culture of *P. vivax*.

The possibility to efficiently expand the CD34^+^ population to generate more reticulocytes coupled with the possibility to cryopreserve those HSPC-derived reticulocytes [7] opens also new perspectives to create stocks of reticulocytes to use as target cells for the establishment of an *in vitro* culture of *P. vivax*.
